# SS18-SSX, the Oncogenic Fusion Protein in Synovial Sarcoma, Is a Cellular Context-Dependent Epigenetic Modifier

**DOI:** 10.1371/journal.pone.0142991

**Published:** 2015-11-16

**Authors:** Sakura Tamaki, Makoto Fukuta, Kazuya Sekiguchi, Yonghui Jin, Sanae Nagata, Kazuo Hayakawa, Sho Hineno, Takeshi Okamoto, Makoto Watanabe, Knut Woltjen, Makoto Ikeya, Tomohisa Kato, Junya Toguchida

**Affiliations:** 1 Department of Tissue Regeneration, Institute for Frontier Medical Sciences, Kyoto University, Kyoto, Japan; 2 Department of Cell Growth and Differentiation, Center for iPS Cell Research and Application, Kyoto University, Kyoto, Japan; 3 Department of Orthopaedic Surgery, Graduate School of Medical Sciences, Nagoya City University, Nagoya, Japan; 4 Department of Orthopaedic Surgery, Graduate School of Medicine, Kyoto University, Kyoto, Japan; 5 Life Science Research Center, Technology Research Laboratory, Shimadzu Corporation, Kyoto, Japan; 6 Department of Life Science Frontiers, Center for iPS Cell Research and Application, Kyoto University, Kyoto, Japan; Johns Hopkins University, UNITED STATES

## Abstract

The prevalence and specificity of unique fusion oncogenes are high in a number of soft tissue sarcomas (STSs). The close relationship between fusion genes and clinicopathological features suggests that a correlation may exist between the function of fusion proteins and cellular context of the cell-of-origin of each tumor. However, most STSs are origin-unknown tumors and this issue has not yet been investigated in detail. In the present study, we examined the effects of the cellular context on the function of the synovial sarcoma (SS)-specific fusion protein, SS18-SSX, using human pluripotent stem cells (hPSCs) containing the drug-inducible *SS18-SSX* gene. We selected the neural crest cell (NCC) lineage for the first trial of this system, induced SS18-SSX at various differentiation stages from PSCs to NCC-derived mesenchymal stromal cells (MSCs), and compared its biological effects on each cell type. We found that the expression of *FZD10*, identified as an SS-specific gene, was induced by SS18-SSX at the PSC and NCC stages, but not at the MSC stage. This stage-specific induction of *FZD10* correlated with stage-specific changes in histone marks associated with the *FZD10* locus and also with the loss of the BAF47 protein, a member of the SWI/SNF chromatin-remodeling complex. Furthermore, the global gene expression profile of hPSC-derived NCCs was the closest to that of SS cell lines after the induction of SS18-SSX. These results clearly demonstrated that the cellular context is an important factor in the function of SS18-SSX as an epigenetic modifier.

## Introduction

The biological phenotype of each type of cancer is defined by genomic and epigenomic alterations that exist in cancer cells, some of which are regarded as “driver” mutations based on their importance in the tumorigenesis of each cancer type [1,2]. One tumor-type-specific driver mutation is a fusion oncogene produced by chromosomal translocations. The prevalence and specificity of unique fusion genes is high in a number of soft tissue sarcomas (STSs), and this is useful for molecular diagnoses and also serves as a tool for identifying therapeutic targets [3,4]. However, in some cases, identical fusion genes have been detected in completely different tumor types, *i*.*e*., *EWSR1*-*ATF1* in clear cell sarcoma (CCS) and angiomatoid fibrous histiocytoma) [5,6], and *ETV6*-*NTRK3* in congenital fibrosarcoma and acute myeloid leukemia [7,8], suggesting that the cellular context of the cell-of-origin of tumors plays an important role in the function of each fusion protein. This issue is also important when suitable therapeutic targets are being searched for among the downstream genes. However, most STSs are origin-unknown tumors; therefore, the cellular context of the cell-of-origin of tumors has not yet been investigated in detail.

Synovial sarcoma (SS) is an origin-unknown STS with a unique fusion gene generated by a specific chromosomal translocation t(X;18)(p11.2;q11.2), which has been detected in more than 95% of tumors [9,10]. This translocation results in the fusion of the *SS18* (also known as *SYT*) gene on chromosome 18 to the *SSX1*, *SSX2*, or *SSX4* gene on the X chromosome, thereby creating the *SS18-SSX* fusion gene [10,11]. Previous studies reported that the SS18-SSX fusion protein functioned as an oncoprotein and played a critical role in the development of SS [12,13]. SS18-SSX consists of the domain for Trithorax group (TrxG) proteins in a part of SS18 and that for polycomb-group (PcG) proteins in a part of SSX; therefore, SS18-SSX may function as a transcriptional regulator even though it has no apparent DNA-binding domain [14–16]. Previous studies have shown that SS18-SSX is involved in chromatin remodeling through associations with TrxG and PcG complexes [17–19].

We previously analyzed the gene expression profiles of SSs along with other types of sarcomas using a genome-wide microarray and found that SS shared an expression profile with the malignant peripheral nerve sheath tumor (MPNST) [20], the cell-of-origin of which is a Schwann cell, a derivative of neural crest cells (NCCs) [21]. Furthermore, proteome analyses of STS revealed that SSs were clustered with MPNST and also with CCS [22], which is another NCC-derived tumor [23]. Although these findings are not conclusive for the neural crest origin of SS, and other cellular lineages may be candidates for its origin, since this is the first study to investigate the effects of the cellular context, we selected the neural crest lineage for further analyses.

The expression of direct-downstream genes may serve as a useful marker for monitoring the function of SS18-SSX in various types of cells. Our genome-wide microarray analysis also identified the *Frizzled homologue 10* (*FZD10*) gene, which is a member of the *Frizzled* family and encodes a putative Wnt receptor, as a gene specifically up-regulated in SS [20]. *FZD10* was previously shown to be expressed at very high levels in nearly all SS tumors and cell lines, but was absent in most normal organs, except for the placenta, or in some cancers arising in other tissues [24]. Additionally, knockdown experiments using siRNA showed that FZD10 was significantly involved in the tumor growth of SS [24]. These findings suggest that *FZD10* is a direct target of SS18-SSX and a suitable indicator for monitoring the function of SS18-SSX in the expression of its target genes.

We herein investigated the role of the cellular context in the function of SS18-SSX. Using human pluripotent stem cells (hPSCs) containing the drug-inducible *SS18-SSX2* gene [25], we performed serial expression analyses of SS18-SSX2 at various differentiation stages from PSCs to NCC-derived mesenchymal stromal cells (MSCs), and showed the cellular context-dependent effects of SS18-SSX on the regulation of its target genes. These results demonstrated the importance of the cellular context for the function of SS18-SSX.

## Materials and Methods

### Ethics statement

Experimental protocols involving human subjects were approved by the Ethics Committee of the Department of Medicine and Graduate School of Medicine, Kyoto University. Written informed consent was provided by each donor.

### Cells and reagents

The human SS cell lines used in this study have been described previously [26]. U2OS and 293T cells were obtained from the American Type Culture Collection (ATCC, Manassas, VA, USA). Human dermal fibroblasts (hDFs) and bone marrow stromal cells (BMSCs) were isolated from donors and maintained in DMEM (4.5 g/l glucose) (Nacalai Tesque, Kyoto, Japan) and MEM Alpha+GlutaMAX (Life Technologies, Carlsbad, CA) supplemented with 10% FBS, respectively. Human embryonic stem cell (hESC) (KhES1) and human induced pluripotent stem cell (hiPSC) (414C2) lines were maintained on SNL feeder cells under previously described culture conditions [25]. mTeSR1 medium (STEMCELL Technology, Vancouver, Canada) was used for the feeder-free culture of hPSCs.

### Establishment of drug-inducible hPSC lines by the PB transposon system

KhES1, the hESC line, containing the FLAG-tagged inducible *SS18-SSX2* gene was established from KhES1 [25] and designated KhES1-FL in this study. KhES1 cells harboring the KW111-Stuffer vector, which express mCherry when treated with doxycycline (DOX) [25], were used as a control cell line and designated KhES1-Control. The hiPSC line 414C2, containing FLAG-tagged inducible *SS18-SSX2*, was also used [25]. We established KhES1 containing 3xHA-tagged *SS18-SSX2*, which was designated KhES1-HA. The entire coding region of the *SS18-SSX2* gene with the 3xHA tag was cloned into the pCR8/GW/TOPO/TA vector (Life Technologies) and transferred into KW111/GW, a derivative of PB-TET containing the rtTA transactivator [27], via the LR clonase reaction, resulting in KW111-3xHA-*SS18-SSX2*, which was then transfected into KhES1 cells, as previously described [25,27]. After expansion, we validated the expression of SS18-SSX2 at the mRNA and protein levels following the administration of DOX (LKT Laboratory, Inc., St. Paul, USA). In order to observe the expression of mCherry in DOX-inducible hPSCs, cells were cultured under feeder-free conditions.

### Induction of hNCCs from hPSCs

Human NCCs (hNCCs) was induced from KhES1 or 414C2 cells as previously described [28]. The efficiency of the induction of hNCCs was evaluated by the fraction of p75^high^ cells and expression of hNCC markers. Drug-inducible hNCCs were maintained in CDM supplemented with SB (Sigma, St. Louis, MO, USA), EGF (R&D System, Minneapolis, USA),) and bFGF (WAKO, Osaka, Japan) on a fibronectin (Millipore, Bedford, CA, USA)-coated dish. The expression of SS18-SSX2 at the mRNA and protein levels was validated 24 h after the DOX treatment at the indicated concentrations.

### Induction of hMSCs from hNCCs

The induction of human MSCs (hMSCs) from hNCC was performed as previously described [28]. The efficiency of the hMSC induction was evaluated based on the expression of hMSC markers (CD73, CD44, CD45, and CD105) and differentiation properties toward osteogenic, chondrogenic, and adipogenic lineages. The expression of SS18-SSX2 at the mRNA and protein levels was validated at each time point after the DOX treatment at the indicated concentrations.

### RNA interference

An siRNA duplex was transfected into SS cells (3 x10^5^ cells) using Lipofectamine 2000 (Life Technologies) at a concentration of 20 nM according to the manufacturer’s instructions. RNA and protein were extracted 72 h after transfection. Two different siRNAs (si*SS18-SSX2* #1 and si*SS18-SSX2* #2) were used to rule out the possibility of an off-target effect. si*SS18-SSX2* #1 was customarily synthesized by Thermo Fisher Scientific (sequences listed in S1 Table) and si*SS18-SSX2* #2 was purchased from Life Technologies (s13506).

### Luciferase assay

DNA fragments of the 5’ flanking regulatory region of the *FZD10* gene were amplified by PCR with Prime STAR DNA polymerase (Takara, Shiga, Japan) and cloned into the luciferase reporter plasmid, PGV-basic (Toyo Ink, Tokyo, Japan). The primers used to amplify each fragment are listed in S1 Table. SYO-1 cells were transfected with each reporter plasmid and phRL-CMV *Renilla*-Luciferase vector (Promega, Madison, WI, USA) using Lipofectamine LTX (Life Technologies) according to the manufacturer’s instructions. Cells were harvested 24 h after transfection and the luciferase assay was performed with the Dual Luciferase Assay Reporter System (Promega) as described previously [29].

### Forced expression of SS18-SSX2

U2OS cells were transfected with pLenti6/V5-DEST/3xHA-*SS18-SSX2* using Lipofectamine LTX according to the manufacturer’s instructions. The pLenti6/V5-DEST/FLAG-*SS18-SSX1* or -*SS18-SSX2* vector was used for lentiviral infection. The lentivirus was produced with ViraPower™ Lentiviral Expression Systems (Life Technologies) according to the manufacturer’s instructions. hDFs and hBMSCs were infected with the viral supernatant containing either the *SS18-SSX1* or *SS18-SSX2* gene.

### Reverse transcription (RT)-PCR and qPCR

Total RNA was isolated from cells using an RNeasy Mini Kit (QIAGEN, Valencia, CA, USA) and the RT reaction was performed using 1 to 2 μg of total RNA with the SuperScript III first-strand synthesis system (Life Technologies) according to the manufacturer’s instructions. The sets of primers used for conventional PCR and qPCR are listed in S1 Table. qPCR was performed in triplicate using SYBR Green reagent (Applied Biosystems, Forester City, CA, USA).

### Western blotting

The preparation of cell lysates and procedures used for SDS-PAGE and blotting were described previously [26]. Immunoreactive bands were detected with Amercham™ ECL™ Prime Western Blotting Detection Reagent (GE Healthcare, Tokyo, Japan) and visualized using BIO-RAD MolecularImager® Chemi-Doc^TM^ XRS+ with Image Lab^TM^ software. The antibodies used in Western blotting are listed in S2 Table.

### Chromatin immunoprecipitation (ChIP) assay

The ChIP assay was performed as previously described [30]. Briefly, cells were incubated with formaldehyde at a final concentration of 1% for 10 min at room temperature to cross-link protein with DNA. The protein-DNA complex was then extracted by lysis buffer (1% SDS; 10 mM EDTA; 50 mM Tris-HCl) and sheared into 300–500 bp fragments using a sonicator. After centrifugation, the supernatants were incubated with antibodies (listed in S2 Table) at 4°C overnight. The next day, Protein G beads (Millipore) were added and centrifuged at 8000 rpm for 1 min to precipitate the complex. After several washing steps, the chromatin-antibody complex was eluted with elution buffer (1% SDS, 0.1 M CH_3_CO_2_Na, 10 mM DTT), and the cross-link between protein and DNA was reversed with 200 mM of NaCl at 65°C overnight. The mixture was treated with 50 μg/ml proteinase K, extracted with phenol/chloroform, and precipitated with ethanol. A qPCR analysis was performed with SYBR Green using primer sets (listed in S1 Table).

### WST-8 assay

Cell viability was measured using the AlamarBlue assay kit (Life Technologies) according to the manufacturer’s protocol. Briefly, cells (5.0 x10^3^/well) were seeded onto a 96-well plate and, after 48 h, 10% AlamarBlue dye was added to each well, followed by a 3-h incubation at 37°C. AlamarBlue fluorescence was assayed at 530 nm and 590 nm using the 2104 EnVision® Xcite Multilabel Reader.

### Fluorescence-activated cell sorting (FACS)

FACS was performed by AriaII (BD, Bedford, MA, USA) according to the manufacturer’s protocol. The antibodies used in FACS were listed in S2 Table.

### cDNA microarray

A microarray analysis was performed according to standard procedures as previously described [25]. Total RNA was prepared using the RNeasy Mini Kit (QIAGEN). cDNA was synthesized using the GeneChip WT (Whole Transcript) Sense Target Labeling and Control Reagents kit as described by the manufacturer (Affymetrix, Santa Clara, CA, USA). Hybridization to GeneChip Human Gene 1.0 ST expression arrays, washing, and scanning were performed according to the manufacturer’s protocol (Affymetrix). Expression values were calculated using the RMA summarization method and the data obtained were analyzed by GeneSpring GX 11.6 (Agilent Technologies, Santa Clara, CA, USA) for a Principle Component Analysis (PCA) and Gene Ontology (GO) analysis. Differentially expressed genes were identified by fold changes. Microarray data have been submitted to the Gene Expression Omnibus (GEO) public database at NCBI with the accession number GSE63895. Data for SS18-SSX2-inducible hPSCs (KhES1 and 414C2) were previously described [25].

### Differentiation of hNCC-derived hMSCs

#### Osteogenic differentiation

Osteogenic differentiation was performed in growth medium supplemented with 0.1 μM dexamethasone (WAKO), 50 μM ascorbic acid (Nacalai Tesque), and 10 mM β-glycerophosphate (Sigma) as previously described [31]. After a 14-day induction, calcium deposits were visualized by Alizarin Red staining.

#### Chondrogenic differentiation

Two-dimensional chondrogenic induction was performed as previously described [32]. Briefly, cells (1.5 x10^5^) were suspended in 5 μl of chondrogenic medium (DMEM/F12 (Life Technologies), 1% (v/v) ITS1 mix (BD), 0.17 mM AA2P (Sigma), 0.35 mM Proline (Sigma), 0.1 μM dexamethasone (WAKO), 0.15% (v/v) glucose (Sigma), 1 mM Na-pyruvate (Sigma), and 2 mM GlutaMax (Life Technologies) supplemented with 40 ng/ml PDGF-BB (R&D System) and 1% (v/v) FBS (Nichirei, Inc., Tokyo, Japan)). They were subsequently transferred to fibronectin-coated 24-well plates (Corning, Inc., NY, USA). One milliliter of the chondrogenic medium was added after 1 h. TGFb3 (R&D System) was subsequently added at 10 ng/ml on days 6 to 10. Differentiation was confirmed on day 10 using Alcian Blue staining.

#### Adipogenic induction

Cells were seeded on 6-well tissue culture dishes at a density of 1.0 x10^6^ cells/well, and adipogenic differentiation was initiated by three cycles of an induction/maintenance culture as previously described [33]. Each cycle consisted of a 3-day culture in induction medium (DMEM (Nacalai Tesque) containing 10% FBS (GE Healthcare), 1 μM dexamethasone (WAKO), 10 μg/ml insulin (Nacalai Tesque), 0.2 mM indomethacin (Nacalai Tesque), and 0.5 mM IBMX (Sigma)), followed by a 3-day culture in maintenance medium (DMEM containing 10% FBS and 10 μg/ml insulin). After an 18-day induction, lipid vacuoles were visualized using Oil Red O staining.

## Results

### Direct regulation of the *FZD10* gene by the SS18-SSX2 fusion protein

**Fig 1:**
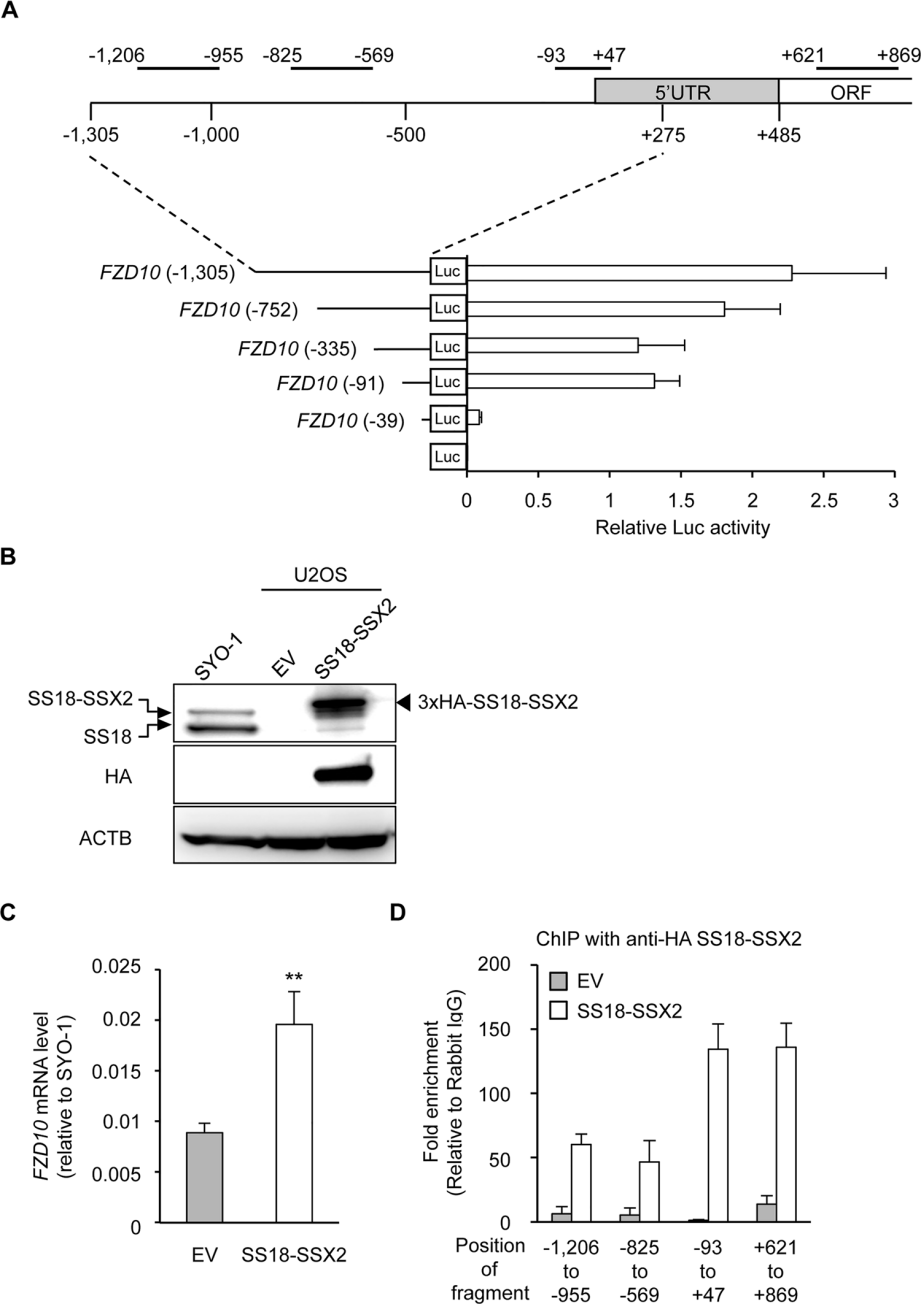
Direct regulation of the *FZD10* gene by the SS18-SSX2 fusion protein. A) Promoter activity in the regulatory region of the *FZD10* gene. The upper panel indicated the 5’ region of the *FZD 10* gene with amplified regions in the ChIP-qPCR analysis. The number indicates relative to the transcription start site, and the positions of the amplified region are: -1206 to -955 bp; -825 to -569 bp; -93 to +47 bp; +621 to +869 bp. The lower panel showed the promoter activities of fragments derived from this 5’ region in SYO-1. Error bars reflect SD in 3 experiments. B) Induction of SS18-SSX2 in U2OS. U2OS was transfected with an empty vector (EV) or 3xHA-tagged *SS18-SSX2*, and 48 h after the transfection, the expression of SS18-SSX2 was analyzed by Western blotting. The SS18-SSX2 and SS18 proteins were detected by an anti-SS18 antibody (top panel), and the 3xHA-SS18-SSX2 protein was detected by an anti-HA antibody (middle panel). C) Induction of *FZD10* expression by SS18-SSX2 in U2OS. The expression of *FZD10* was analyzed by RT-qPCR. Expression levels were normalized to those of human *ACTB* and calculated as fold changes relative to SYO-1. Error bars reflect SD in 3 experiments. **, p<0.01 by the *t*-test. D) Binding of SS18-SSX2 to the *FZD10* locus. A ChIP assay with an anti-HA antibody and RT-qPCR were performed. The values indicate relative to rabbit IgG. Error bars reflect SD in 3 experiments.

The *FZD10* gene has been identified as a downstream gene of SS18-SSX by microarray analyses [20], and siRNA knockdown experiments also showed that the expression of the *FZD10* gene was regulated by SS18-SSX2 (S1A and S1B Fig). We performed a luciferase assay using reporter constructs containing the *FZD10* upstream region in the FZD10-positive SS cell line (SYO-1) in order to identify the transcriptional regulatory region in the *FZD10* gene (Fig 1A). Although transcriptional activity was decreased by the truncation of the -1305 to -336 fragment, it disappeared when the region between -91 and -40 was truncated (Fig 1A), indicating that this region was important for the basal transcriptional activity of *FZD10*.

An expression vector containing the *SS18-SSX2* gene was introduced into several FZD10-negative cells in order to determine whether SS18-SSX2 induced the expression of the *FZD10* gene. In U2OS, the expression of transfected SS18-SSX2 was confirmed at the protein level (Fig 1B), which showed values higher than those in SYO-1. The expression of the *FZD10* gene was induced by SS18-SSX2 at the same time in U2OS (Fig 1C), and ChIP analyses showed the binding of SS18-SSX2 to the core promoter region of the *FZD10* gene identified by the reporter assay (Fig 1A and 1D). These results indicated that SS18-SSX2 regulated the expression of the *FZD10* gene by binding to this region. However, the introduction of SS18-SSX2 failed to induce the *FZD10* gene in other types of cells, such as hDFs or hBMSCs (S1C Fig). These results suggested that the regulation of *FZD10* by SS18-SSX required an appropriate cellular context.

### Cell type-dependent effects of SS18-SSX on the expression of *FZD10*

In order to investigate the cell type-dependent effects of SS18-SSX in more detail, we induced SS18-SSX2 at various differentiation stages, and compared its effects on the expression of the *FZD10* gene. KhES1-FL as well as KhES1-Control cells differentiated into hNCCs (KhES1-NCC-FL and KhES1-NCC-Control) and then into hMSCs (KhES1-MSC-FL and KhES1-MSC-Control) as previously described [28]. The properties of these differentiated cells were confirmed by the expression of hNCC markers (S2A and S2B Fig) or hMSC markers (positive for CD73, 105, and 44, and negative for CD45) (S2C and S2D Fig). Identical experiments were performed starting from KhES1-HA cells, and the properties of KhES1-MSC-HA cells were also confirmed (S2E Fig). Furthermore, the induced hMSCs successfully differentiated into osteogenic, chondrogenic, and adipogenic lineages (S3A–S3C Fig).

**Fig 2:**
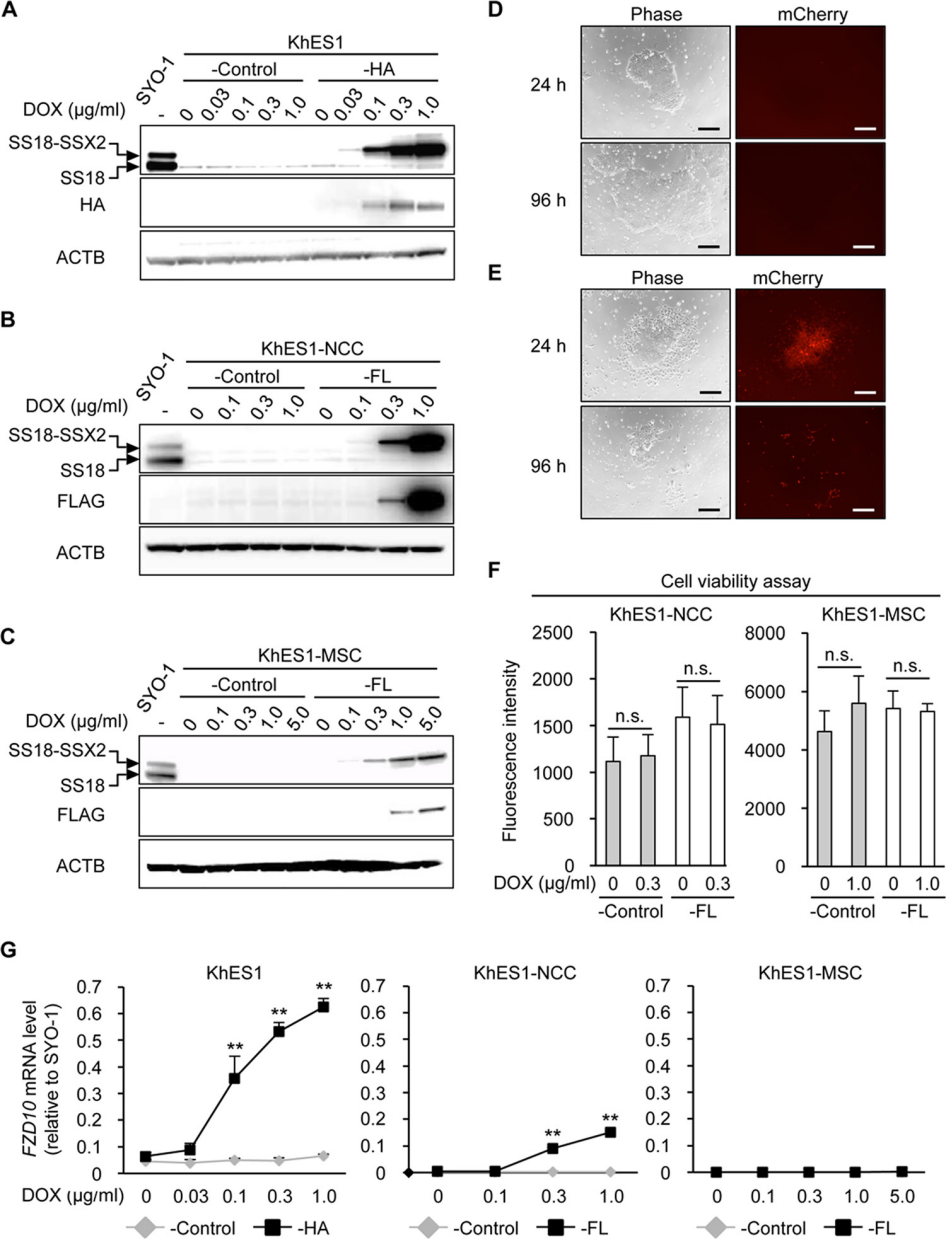
Induction of SS18-SSX2 in hESCs, hNCCs, and hNCC-derived MSCs. A-C) DOX dose-dependently induced the SS18-SSX2 protein in KhES1-HA (A), KhES1-NCC-FL (B), and KhES1-MSC-FL (C) cells. Cells with Stuffer (-Control) and SS18-SSX2 were treated with the indicated concentrations of DOX for 24 h, and the expression of SS18-SSX2 was analyzed by Western blotting. The SS18-SSX2 and SS18 proteins were detected using an anti-SS18 antibody (top panel), and the 3xHA-SS18-SSX2 or FLAG-SS18-SSX2 protein was detected by an anti-HA or anti-FLAG antibody (middle panel). D and E) Morphology (left panels) and expression of mCherry (right panels) in KhES1-HA cells treated with 0 (D) or 0.3 (E) μg/ml of DOX for 24 and 96 h. Scale bar, 200 μm. F) Effects of SS18-SSX2 on the cell viability of KhES1-NCC-FL and KhES1-MSC-FL cells. Cells with Stuffer (-Control) or SS18-SSX2 were treated with the indicated concentrations of DOX for 48 h, and cell viability was measured using the AlamarBlue assay. n.s. means not significant. Error bars reflect SD in 4 experiments. G) Induction of *FZD10* expression by SS18-SSX2 in KhES1-HA, KhES1-NCC-FL, and KhES1-MSC-FL cells. Cells with Stuffer (-Control) or SS18-SSX2 were treated with the indicated concentrations of DOX for 24 h, and the expression of *FZD10* was analyzed by RT-qPCR. Expression levels were normalized to those of human *ACTB* and calculated as fold changes relative to SYO-1. Error bars reflect SD in 3 experiments. **, p<0.01 by the *t*-test.

The DOX treatment successfully induced SS18-SSX2 at the mRNA and protein levels in a dose-dependent manner in three types of cells (Figs 2A–2C and S4A). However, the induction level of SS18-SSX2 was lower in KhES1-MSC-FL cells than in KhES1-HA or KhES1-NCC-FL cells even at a high concentration of DOX (Figs 2A–2C and S4A). In order to accurately compare the effects of SS18-SSX among cell lines, the levels of the SS18-SSX2 protein induced in each cell line was expected to be similar to that induced in the human SS cell line. Therefore, we compared the SS18-SSX2 protein levels induced in KhES1 cells, KhES1-NCCs, and KhES1-MSCs with those in SYO-1 cells, and then determined the concentration of DOX for each cell type (0.1, 0.3, and 1.0 μg/ml for KhES1-HA, KhES1-NCC-FL, and KhES1-MSC-FL cells, respectively) (S4B Fig).

SS18-SSX2-expressing hESCs exhibited morphological changes from the edges of the colonies 24 h after being induced and gradually died (Fig 2D and 2E), whereas no morphological changes were observed in KhES1-NCCs or KhES1-MSCs after the DOX treatment. The WST-8 assay revealed that SS18-SSX2 did not affect the cell viability of these cells, at least 48 h after the induction (Fig 2F). Regarding the induction of *FZD10*, the ectopic expression of SS18-SSX2 significantly induced the expression of *FZD10* in KhES1-HA and KhES1-NCC-FL cells, but not in KhES1-MSC-FL cells (Fig 2G). The prolonged treatment of KhES1-MSC-FL cells with DOX increased the level of the SS18-SSX2 in a time-dependent manner (S4C and S4D Fig), whereas it failed to induce the *FZD10* gene (S4E Fig).

Taken together, our serial expression analyses of SS18-SSX2 in different types of cells from the pluripotent stage to hNCC-derived MSCs clearly demonstrated that SS18-SSX had cell type-dependent effects, and the cellular context of hESC-derived NCCs appeared to be permissive for the expression of SS18-SSX in terms of cell viability and induction of the *FZD10* gene.

### Cell type-dependent effects of SS18-SSX on global gene expression profiles

**Fig 3:**
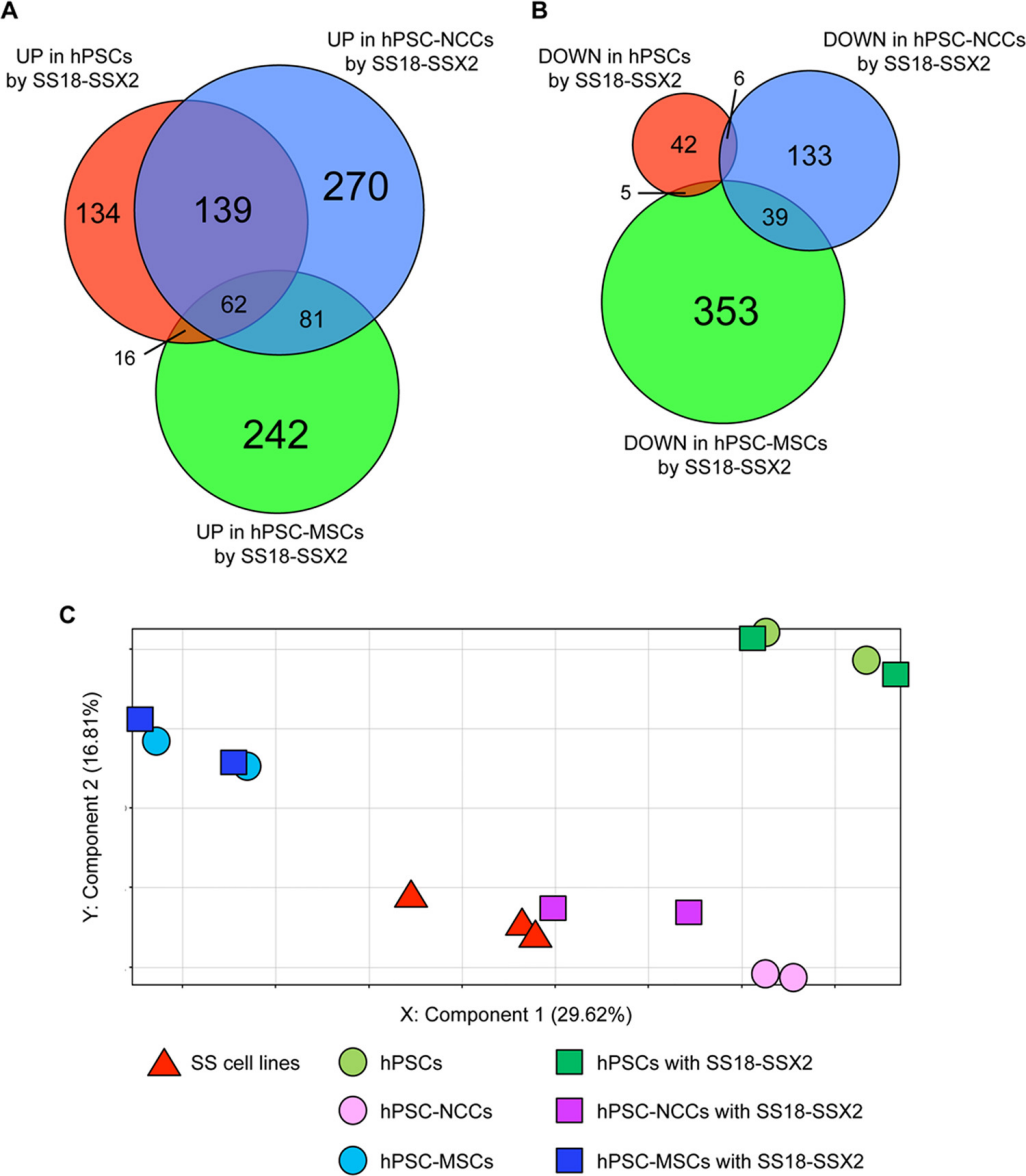
Global gene expression profiles of hPSCs, hPSC-NCCs, and hPSC-MSCs with or without the induction of SS18-SSX2. A and B) Venn-diagram showing up- (A) and down-regulated (B) genes in hPSCs, hPSC-NCCs, and hPSC-MSCs with the induction of SS18-SSX2. mRNA was extracted from each cell line 24 h after the DOX treatment. Each gene showed a fold change > 2.0 with or without the induction of SS18-SSX2. The numbers of up- and down-regulated genes are shown in the diagram. C) PCA of hPSCs, hPSC-NCCs, and hPSC-MSCs with or without the induction of SS18-SSX2, and SS cell lines. The cell lines and conditions used in this experiment are described in S3 Table. mRNA was extracted from each cell line 12 h after the DOX treatment.

In order to determine whether the cell type-dependent effects of SS18-SSX were specific to the *FZD10* gene, we analyzed genome-wide expression profiles in hPSCs, hPSC-NCCs, and hPSC-MSCs with or without the induction of SS18-SSX2 (S3 Table). Genes that were up- or down-regulated more than two-fold by SS18-SSX2 in each type of cell were identified and categorized into several groups based on their specificities (Fig 3A and 3B). As expected, *FZD10* was categorized into genes up-regulated in hPSCs and hPSC-NCCs, but not into those in hPSC-MSCs (S4 Table). Among the 552 genes up-regulated in hPSC-NCCs, 139 (25.2%) were categorized into this group (Fig 3A and S4 Table), suggesting that a large number of genes behaved in a similar manner to *FZD10*. GO term analyses identified “sequence-specific DNA binding transcription factor activity” as the top-ranking feature of these genes and also suggested involvement in the developmental process such as “multicellular organismal development” and “nervous system development” (S5 Table). A number of one-stage-specific genes were also identified. A total of 134/351 (38.2%), 270/552 (48.9%), and 242/401 (60.3%) genes were up-regulated in hPSCs, hPSC-NCCs, and hPSC-MSCs, respectively (Fig 3A and S6–S8 Tables). Genes that were down-regulated by SS18-SSX2 showed greater cell-type specificity than up-regulated genes. Cell type-specific down-regulated genes were 42/53 (79.2%) in hPSCs, 133/178 (74.7%) in hPSC-NCCs, and 353/397 (88.9%) in hPSC-MSCs (Fig 3B and S9–S11 Tables). These results clearly showed that SS18-SSX regulated different downstream genes depending on the cell type, suggesting that the cell context is an important determinant for its function.

Furthermore, PCA revealed that hPSCs, hPSC-NCCs, and hPSC-MSCs showed clearly distinct expression profiles, which were also different from those of SS cell lines (Fig 3C). The profile of hPSC-NCCs, but not that of hPSCs or hPSC-MSCs, became closer to that of SS cell lines with the induction of SS18-SSX2 (Fig 3C).

### Cell type-dependent function of SS18-SSX2 as an epigenetic modifier

Recent studies showed the involvement of SS18-SSX in chromatin remodeling [17,34,35], and these findings prompted us to investigate the cell type-dependent function of SS18-SSX as an epigenetic modifier. Modifications of histones associated with the regulatory region of the *FZD10* locus in endogenous *FZD10* negative-hDFs and -positive SYO-1 cells were initially analyzed (S5A and S5B Fig). Histone modifications of hDFs showed a high level of repressive mark (H3K27me3) through the analyzed region (S5A Fig), whereas the level of active marks (H3K4me3 and H3Ac) was high in SYO-1 (S5B Fig). These results indicated that the status of histones in the analyzed region correlated with the expression of *FZD10*. Each type of histone modification at the *FZD10* locus was then analyzed in KhES1 cells, KhES1-NCCs, and KhES1-MSCs with and without the induction of SS18-SSX2.

**Fig 4:**
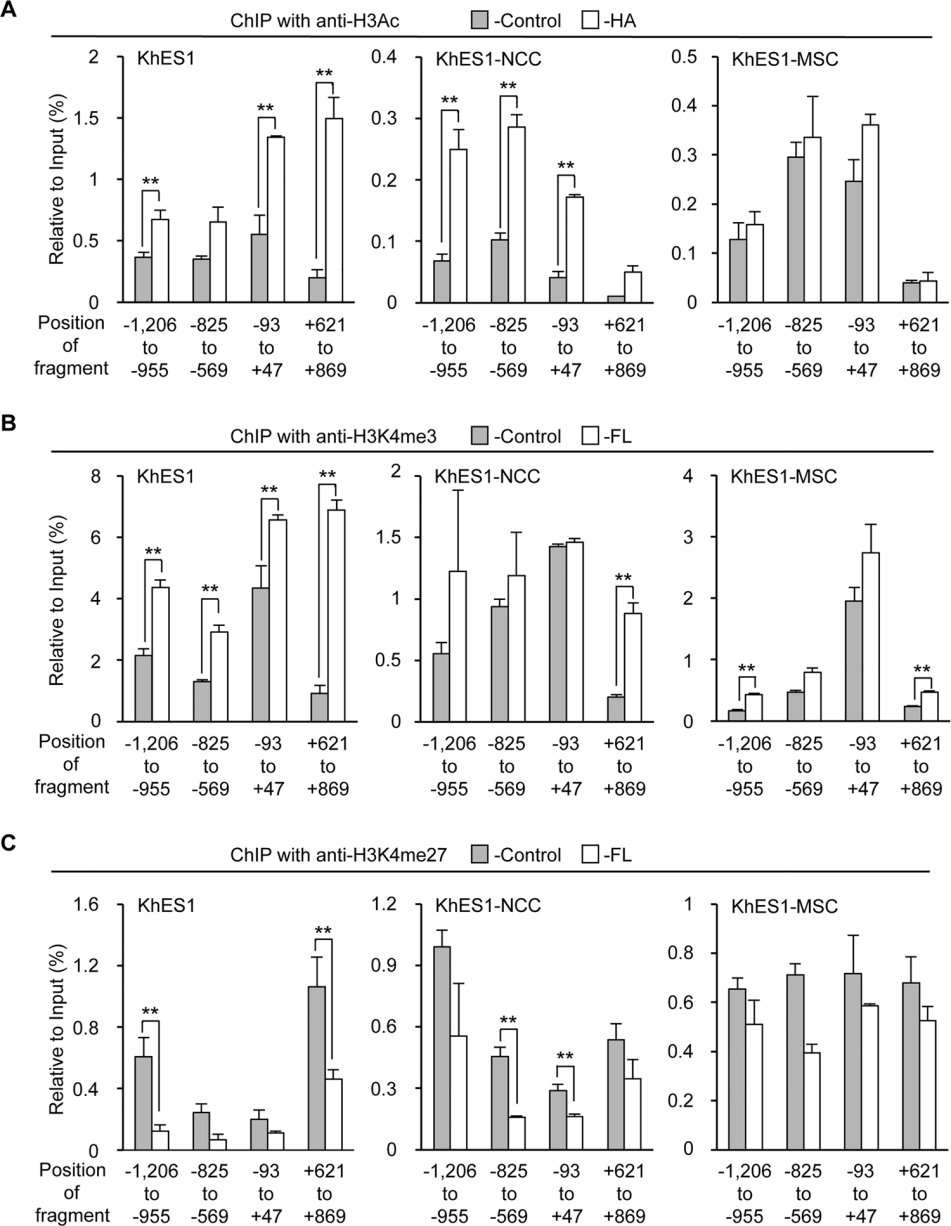
Cell type-dependent effects of SS18-SSX2 on histone modifications at the *FZD10* locus. A-C) Changes in histone modifications at the *FZD10* locus by SS18-SSX2 in KhES1-HA, KhES1-NCC-FL, and KhES1-MSC-FL cells. Cells with Stuffer (-Control) and SS18-SSX2 were treated for 24 h with DOX (0.1, 0.3, and 1.0 μg/ml for KhES1-HA, KhES1-NCC-FL, and KhES1-MSC-FL cells, respectively). The levels of H3Ac (A), H3K4me3 (B), and H3K27me3 (C) were analyzed by ChIP-qPCR. The values indicate relative to the input. Error bars reflect SD in 3 experiments. **, p<0.01 by the *t*-test.

In the case of H3Ac, SS18-SSX2 clearly increased this modification in khES1-HA and KhES1-NCC-FL cells, whereas the amount of H3Ac appeared to be same even after the induction of SS18-SSX2 in KhES1-MSC-FL cells (Fig 4A). The modification of H3K4me3 was also clearly enhanced by SS18-SSX2 in KhES1 cells (Fig 4B). This enhancement was limited, but still observed in KhES1-NCCs, whereas no marked change was observed in KhES1-MSCs (Fig 4B). The amount of the repressive modification, H3K27me3, was reduced by SS18-SSX in KhES1 cells and KhES1-NCCs, but remained at a higher level in KhES1-MSCs (Fig 4C). Since the level of SS18-SSX2 induced was similar, the difference observed in histone modifications by SS18-SSX2 may have been due to the different cellular context in each cell type.

### Relationship between BAF47 levels and the induction of *FZD10*

**Fig 5:**
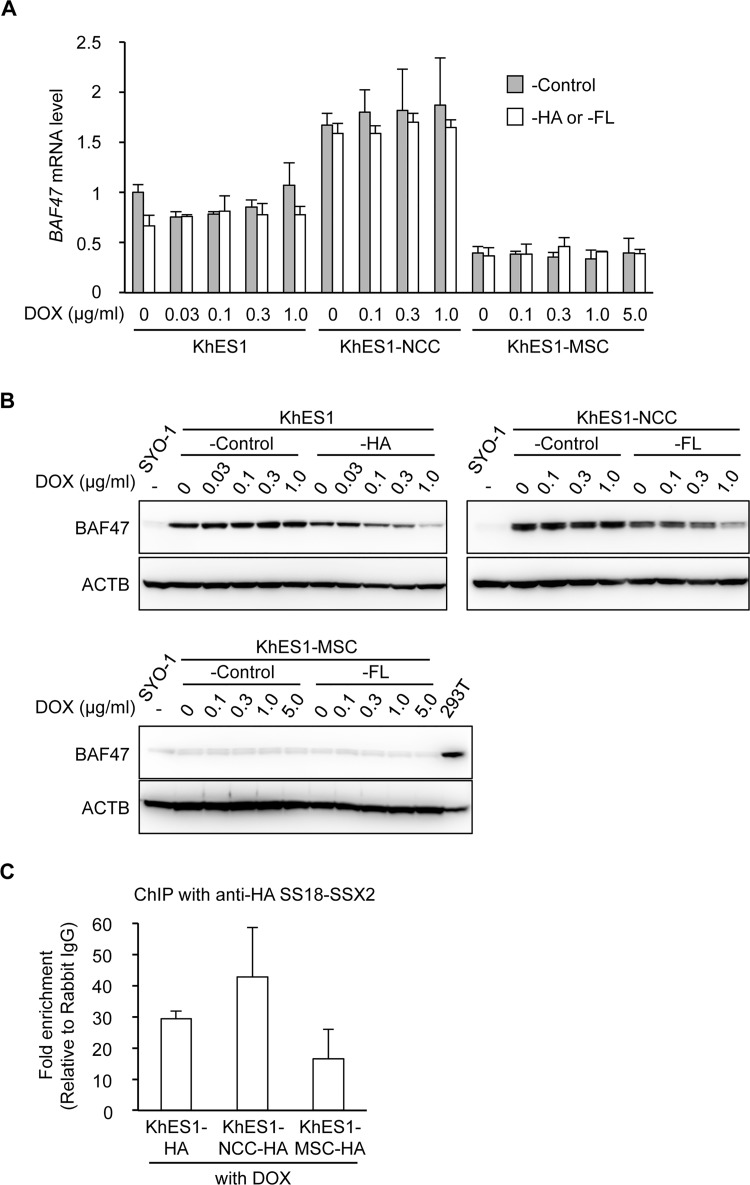
Cell type-dependent differences in BAF complexes. A and B) The mRNA (A) and protein (B) expression of BAF47 in KhES1-HA, KhES1-NCC-FL, and KhES1-MSC-FL cells. Cells with Stuffer (-Control) and SS18-SSX2 cells were treated with the indicated concentrations of DOX for 24 h, and the expression of BAF47 was analyzed. A) mRNA expression analysis using RT-qPCR. Expression levels were normalized to those of human *ACTB*. Error bars reflect SD in 3 experiments. B) Protein expression analysis by Western blotting. The BAF47 protein was detected using an anti-BAF47 antibody. 293T cells were used as a positive control. C) Recruitment of SS18-SSX2 to the *FZD10* core promoter region (from -93 to +47 bp) in KhES1-HA, KhES1-NCC-HA, and KhES1-MSC-HA cells. Cells were treated with DOX (0.1, 1.0, and 3.0 μg/ml for KhES1-HA, KhES1-NCC-HA, and KhES1-MSC-HA cells, respectively) for 24 h. A ChIP assay with an anti-HA antibody and RT-qPCR were performed. The values indicate relative to rabbit IgG. Error bars reflect SD in 3 experiments.

A recent study demonstrated that SS18-SSX participated in the protein complex consisting of Brg1 and multiple Brg1-associated factors (BAF) by replacing SS18 [18]. As a result, BAF47, one of the core members of the BAF complex, was eliminated from the complex and subsequently degenerated by proteasomes. In order to investigate the involvement of BAF47 in our system, the effects of SS18-SSX2 on the expression of BAF47 were analyzed. The expression level of the *BAF47* gene was higher in KhES1-NCC-FL cells than in KhES1-HA cells and significantly lower in KhES1-MSC-FL cells (Fig 5A, under the no DOX condition). The induction of SS18-SSX2 in these cell lines had negligible effects on the mRNA expression of *BAF47* (Fig 5A). However, protein expression was markedly decreased in KhES1-HA in a DOX-dose dependent manner, namely, a SS18-SSX2-dose dependent manner (Fig 5B). Since mRNA levels were stable (Fig 5A), this decrease may have been due to enhancements in the degradation process. Similar reductions were observed in the BAF47 protein in KhES1-NCC-FL, whereas no marked change was noted in KhES1-MSC-FL (Fig 5B). Identical stage-dependent data were obtained in cell lines with a different tag (S6A Fig), which supported this stage-dependent difference being caused by the stage-dependent function of SS18-SSX.

We also examined the recruitment of SS18-SSX2 to the *FZD10* core promoter in the three types of cells. Although no significant differences were noted, the binding affinity of SS18-SSX2 was lower in KhES1-MSC-HA cells than in KhES1-HA and KhES1-NCC-FL cells, which was related to the stage-specific induction of *FZD10* (Figs 5C and S6B and S6C). These results suggested that a difference in the transcriptional regulatory complex in each cell type was an important cellular context determining the function of SS18-SSX as an epigenetic modifier.

## Discussion

Recent *in vivo* and *in vitro* studies have elucidated the molecular mechanisms underlying SS development, particularly concerning the involvement of SS18-SSX. Although the SS18-SSX fusion protein has no apparent DNA-binding domain, it has been suggested to play a role in chromatin remodeling through an association with TrxG and/or PcG complexes [12,17,18,34,35]. Su *et al*. revealed that SS18-SSX2 bridged ATF2 and TLE1, which is a member of the TLE family of proteins with co-repressor activity, and generated a suppressive complex on ATF2 target genes [17]. On the other hand, Kadoch and Crabtree proposed a model for transcriptional activation, in which SS18-SSX disrupts the normal architecture of the BAF complex by replacing SS18 and eliminating BAF47 from the complex, thereby reversing the polycomb-mediated repression of the *SOX2* gene [18]. In both mechanisms, SS18-SSX requires partners to exert its function, which may differ in each gene and also in each cell type.

Loss-of-function experiments using SS cell lines and gain-of-function approaches using normal cells identified several target genes of SS18-SSX, such as *IGF2* and *EGR1*, and showed the important role of SS18-SSX in cell growth and focal adhesion [35–40]. However, the gene set identified in these *in vitro* experiments did not necessarily match those found by the expression profiles of SS tumors [13,41,42]. These findings implied that the contexts of cells used in previous experiments were not fully appropriate for understanding the function of SS18-SSX. Therefore, we herein investigated the impact of the cellular context on the function of SS18-SSX. Based on our and others’ previous studies, we selected cells in the neural crest lineage as tentative target cells of SS18-SSX, and induced SS18-SSX2 at the PSC, NCC, and NCC-derived MSC stages. As a result, the expression of *FZD10*, a direct target of SS18-SSX2, was detected in hPSCs and hPSC-NCCs, but not in hPSC-MSCs with the induction of SS18-SSX2. Furthermore, SS18-SSX2 had cell type-dependent effects on cell viability, which was consistent with previous findings showing growth-suppressive effects in certain cell types. *Nagai et al*. reported differences in the transforming activity of SS18-SSX1 between 3Y1 rat fibroblasts and NIH 3T3 mouse fibroblasts [12], which may be related to the up-regulation of p21 by SS18-SSX1 in the latter, but not in the former cells [43].

We observed a similar cell type-related induction of the *FZD10* gene by SS18-SSX2, and, importantly, this stage-specific induction correlated with the stage- specific change in histone modifications. SS18-SSX2 changed the histone marks of the *FZD10* locus into an active state by reducing H3K27me3, and increasing H3K4me3 and H3Ac in hESCs and hNCCs, but not in hNCC-derived MSCs. In other words, the cellular context had a prominent impact on the function of SS18-SSX as an epigenetic modifier.

The responsible factors of the cellular context for determining the effects of oncogenic events currently remain unclear. In the case of the *EWSR1-ATF1* fusion gene, the context of the target cells of CCS determined the expression of the *MITF* gene, which is the key transcription factor for the melanocytic phenotype of CCS [6,44]. Although we have not yet elucidated the underlying mechanism in SS, the presence of BAF47 appeared to be an important factor for determining the effects of SS18-SSX. In the present study, we demonstrated that SS18-SSX2 decreased the expression level of the BAF47 protein in hESCs and hESC-NCCs, which is consistent with previous findings [18]. However, the endogenous expression of BAF47 was markedly lower in hESC-MSCs than in hESCs or hESC-NCCs. BAF47 is a member of the ATP-dependent SWI/SNF chromatin-remodeling complex and its expression was previously shown to be reduced in SS tissues [45]. Mammalian BAF complexes are considered to be combinationally assembled from several subunits during development and acquire specific functions in biological processes, including the maintenance of pluripotency and neuronal differentiation [46,47]. Therefore, the content of the BAF complex associated with the *FZD10* gene may vary in cell stages, which may cause the cellular context-dependent regulation of this gene by SS18-SSX. Precise biochemical analyses of the members of the complex associated with the promoter of *FZD10* in each cell type will provide more concrete evidence for this cellular context specificity, and we are currently investigating this issue.

Although recent studies have advocated several possible cellular origins of SS, including multipotent stem cells and precursors of a muscle lineage, the cell-of-origin of SS still remains controversial [13,48–50]. Our results showed that the induction of SS18-SSX2 altered the global gene expression of hNCCs to be closer to those of SS cell lines; therefore, hNCC may serve as the origin of SS. Although we employed the neural crest lineage in this study, our inducible system is applicable to other lineages in order to identify the cells-of-origin of SS. In addition, the cellular context-specific function of fusion proteins represents an important issue in the search for target molecules for the treatment of SS, and our inducible system will be a powerful tool for investigating this issue.

## Supporting Information

S1 Fig Direct regulation of the *FZD10* gene by the SS18-SSX2 fusion protein. A) Effects of siRNA against *SS18-SSX2*. SS cell lines (SYO-1 and 1273/99) were transfected with control siRNA (si-*Ctrl*), si-*SS18-SSX2* #1, or si-*SS18-SSX2* #2, and the expression of the SS18-SSX2 protein was analyzed 72 h after the transfection by Western blotting using an anti-SS18 antibody. B) Downregulation of *FZD10* expression by the knockdown of *SS18-SSX2*. The expression of *FZD10* was analyzed by RT-qPCR. Expression levels were normalized to those of human *ACTB* and calculated as fold changes relative to cells transfected with si-*Ctrl*. Error bars reflect SD in 3 experiments. *, p<0.05 by the *t*-test. C) Induction of *FZD10* by SS18-SSX in hDFs and hBMSCs. Cells were infected with pLenti6/V5-DEST-EV, -*SS18-SSX1*, or -*SS18-SSX2*, and RNA was extracted 48 and 96 h after the infection. The mRNA expression of *SS18-SSX1* and *SS18-SSX2*, and *FZD10* was analyzed by RT-qPCR. NT; non-treated.
(PDF)

S2 Fig Characterization of KhES1-NCCs and KhES1-MSCs. A) Induction efficiency of NCCs from KhES1-FL cells. After the neural crest induction, cells were stained with an anti-p75 antibody and the p75^high^-positive population was analyzed by FACS. B) Expression of neural crest-specific markers in KhES1-FL and KhES1-NCC-FL cells. The mRNA expression of hNCC markers (*SOX10*, *TFAP2A*, *PAX3*, and *NGFR*) was analyzed by RT-qPCR in cells with SS18-SSX2 without the DOX treatment. Expression levels were normalized to those of human *ACTB* and calculated as fold changes relative to KhES1-FL cells. Error bars reflect SD in 3 experiments. C-E) Expression of surface markers in hMSC cells. After the induction of hMSCs, the expression of each CD antigen in KhES1-MSC-Control (C), KhES1-MSC-FL (D), and KhES1-MSC-HA (E) cells was analyzed by FACS.
(PDF)

S3 Fig Differentiation properties of KhES1-MSCs toward osteogenic, chondrogenic, and adipogenic lineages. A-C) KhES-MSC-Control, KhES1-MSC-FL, and KhES1-MSC-HA cells were induced toward osteogenic (A), chondrogenic (B), or adipogenic (C) lineages. Osteogenic induction (OI), chondrogenic induction (CI), and adipogenic induction (AI) were performed as described in the Materials and Methods section, and were evaluated by Alizarin Red staining on day 14, Alcian Blue staining on day 10, and Oil Red O staining on day 18, respectively. hMSCs were cultured during the induction periods in hMSC medium as a negative control (CT). Scale bar, 200 μm in OI and 50 μm in AI.
(PDF)

S4 Fig Induction of SS18-SSX2 in hESCs, hNCCs, and hNCC-derived MSCs. A) DOX dose-dependently induced *SS18-SSX2* mRNA in KhES1-HA, KhES1-NCC-FL, and KhES1-MSC-FL cells. Cells with Stuffer (-Control) and SS18-SSX2 were treated with the indicated concentrations of DOX for 24 h, and the expression of *SS18-SSX2* was analyzed by RT-qPCR. Expression levels were normalized to those of human *ACTB* and calculated as fold changes relative to SYO-1. Error bars reflect SD in 3 experiments. B) Comparison of SS18-SSX2 expression levels among KhES1-HA, KhES1-NCC-FL, and KhES1-MSC-FL cells. Cells with Stuffer (-Control) and SS18-SSX2 were treated with the indicated concentrations of DOX for 24 h, and the expression of SS18-SSX2 was analyzed by Western blotting. The SS18-SSX2 and SS18 proteins were detected using an anti-SS18 antibody. C and D) The time-dependent induction of SS18-SSX2 at mRNA (C) and protein (D) levels in KhES1-MSC-FL cells. Cells with Stuffer (-Control) and SS18-SSX2 were treated with 1.0 μg/ml of DOX for the indicated periods. C) RT-qPCR; Expression levels were normalized to those of human *ACTB* and calculated as fold changes relative to SYO-1. Error bars reflect SD in 3 experiments. D) Western blotting; The SS18-SSX2 and SS18 proteins were detected by an anti-SS18 antibody (top panel), and the FLAG-SS18-SSX2 protein was detected using an anti-FLAG antibody (middle panel). E) Induction of *FZD10* expression by SS18-SSX2 in KhES1-MSC-FL cells. Cells with Stuffer (-Control) and SS18-SSX2 were treated with 1.0 μg/ml of DOX for the indicated periods. The expression of *FZD10* was analyzed by RT-qPCR. Expression levels were normalized to those of human *ACTB* and calculated as fold changes relative to SYO-1. Error bars reflect SD in 3 experiments.
(PDF)

S5 Fig Histone modifications at the *FZD10* locus in fibroblasts and SS cells. A and B) Modifications of histones associated with 5’ regions in the *FZD10* locus of hDF (A) and SYO-1 (B) cells. H3K4me3, H3Ac, and H3K27me3 levels were analyzed by ChIP-qPCR. The values indicate relative to the input. Error bars reflect SD in 3 experiments.
(PDF)

S6 Fig Relationship between BAF47 levels and the induction of *FZD10*. A) Effects of SS18-SSX2 on BAF47 expression levels. KhES1-FL, KhES1-NCC-HA, and KhES1-MSC-HA cells were treated with the indicated concentrations of DOX for 24 h, and the expression of BAF47 was analyzed by Western blotting. The BAF47 protein was detected using an anti-BAF47 antibody. B and C) Induction of *SS18-SSX2* (B) and *FZD10* (C) mRNA in KhES1-NCC-HA and KhES1-MSC-HA cells. Cells with Stuffer (-Control) and SS18-SSX2 were treated with the indicated concentrations of DOX for 24 h, and the expression of *SS18-SSX2* (B) and *FZD10* (C) was analyzed by RT-qPCR. Expression levels were normalized to those of human *ACTB* and calculated as fold changes relative to SYO-1. Error bars reflect SD in 3 experiments. Error bars reflect SD in 3 experiments. **, p<0.01 by the *t*-test.
(PDF)

S1 Table Primer and siRNA sequences used in this study. (PDF)

S2 Table Antibodies used in this study. (PDF)

S3 Table Cell lines and conditions for gene expression profiling. (PDF)

S4 Table List of genes up-regulated in hPSCs and hPSC-NCCs, but not in hPSC-MSCs by SS18-SSX2 (>2.0 fold). (XLSX)

S5 Table GO term of genes up-regulated in hPSCs and hPSC-NCCs by SS18-SSX2. (XLSX)

S6 Table List of genes specifically up-regulated in hPSCs, but not in hPSC-NCCs or hPSC-MSCs by SS18-SSX2 (>2.0 fold). (XLSX)

S7 Table List of genes specifically up-regulated in hPSC-NCCs, but not in hPSCs or hPSC-MSCs by SS18-SSX2 (>2.0 fold). (XLSX)

S8 Table List of genes specifically up-regulated in hPSC-MSCs, but not in hPSCs or hPSC-NCCs by SS18-SSX2 (>2.0 fold). (XLSX)

S9 Table List of genes specifically down-regulated in hPSCs, but not in hPSC-NCCs or hPSC-MSCs by SS18-SSX2 (>2.0 fold). (XLSX)

S10 Table List of genes specifically down-regulated in hPSC-NCCs, but not in hPSCs or hPSC-MSCs by SS18-SSX2 (>2.0 fold). (XLSX)

S11 Table List of genes specifically down-regulated in hPSC-MSCs, but not in hPSCs or hPSC-NCCs by SS18-SSX2 (>2.0 fold). (XLSX)
