# Supplementary figures and images for: SS18-SSX, the Oncogenic Fusion Protein in Synovial Sarcoma, Is a Cellular Context-Dependent Epigenetic Modifier
Source: PLoS One. 2015 Nov 16;10(11):e0142991. doi: 10.1371/journal.pone.0142991 (PMC4646489; doi:10.1371/journal.pone.0142991)

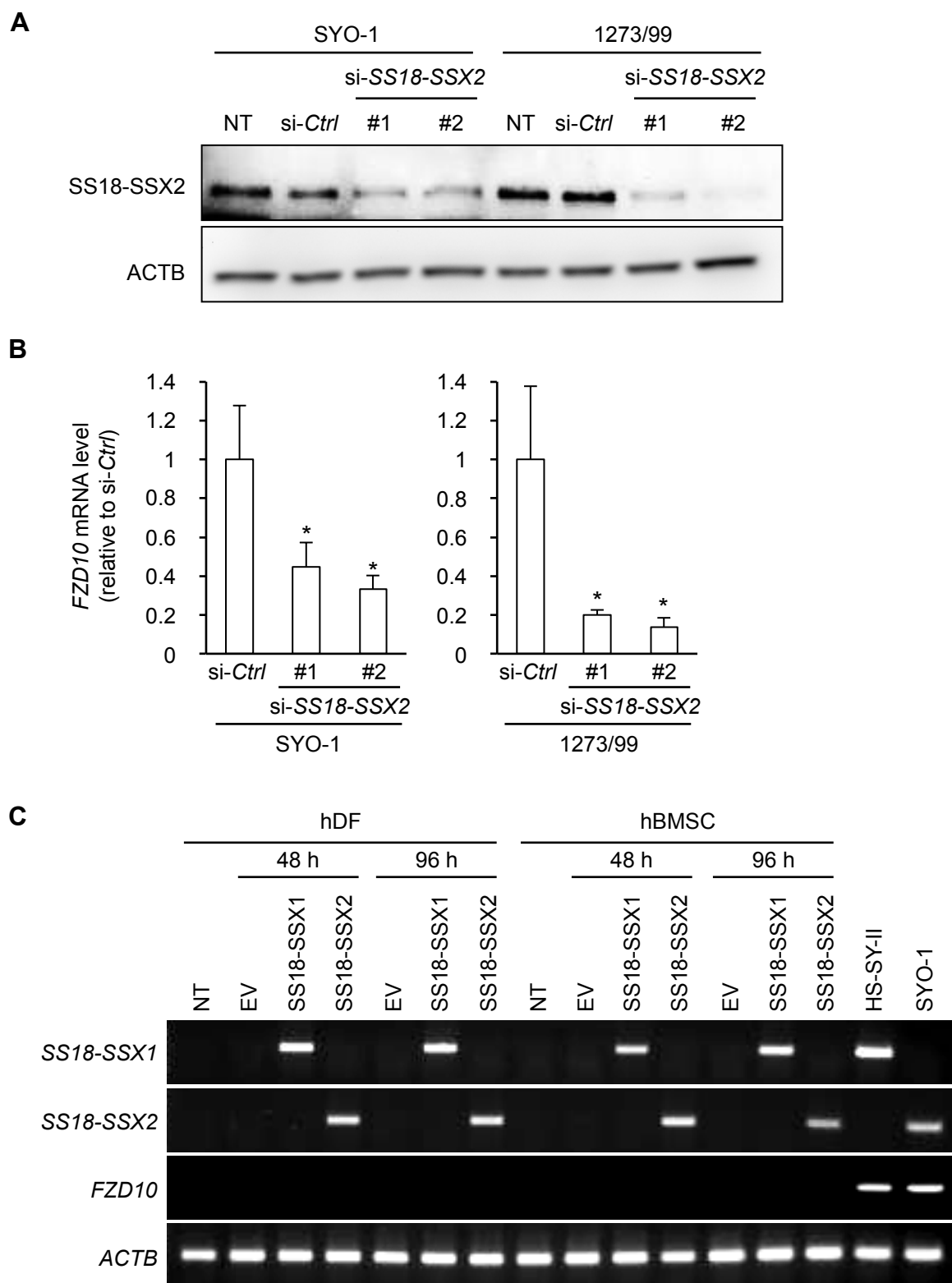

**S1 Fig. Direct regulation of the *FZD10* gene by the SS18-SSX2 fusion protein.**

Supplement: S1 Fig — A) Effects of siRNA against SS18-SSX2. SS cell lines (SYO-1 and 1273/99) were transfected with control siRNA (si-Ctrl), si-SS18-SSX2 #1, or si-SS18-SSX2 #2, and the expression of the SS18-SSX2 protein was analyzed 72 h after the transfection by Western blotting using an anti-SS18 antibody. B) Downregulation of FZD10 expression by the knockdown of SS18-SSX2. The expression of FZD10 was analyzed by RT-qPCR. Expression levels were normalized to those of human ACTB and calculated as fold changes relative to cells transfected with si-Ctrl. Error bars reflect SD in 3 experiments. *, p<0.05 by the t-test. C) Induction of FZD10 by SS18-SSX in hDFs and hBMSCs. Cells were infected with pLenti6/V5-DEST-EV, -SS18-SSX1, or -SS18-SSX2, and RNA was extracted 48 and 96 h after the infection. The mRNA expression of SS18-SSX1 and SS18-SSX2, and FZD10 was analyzed by RT-qPCR. NT; non-treated. (PDF) [file pone.0142991.s001.pdf]

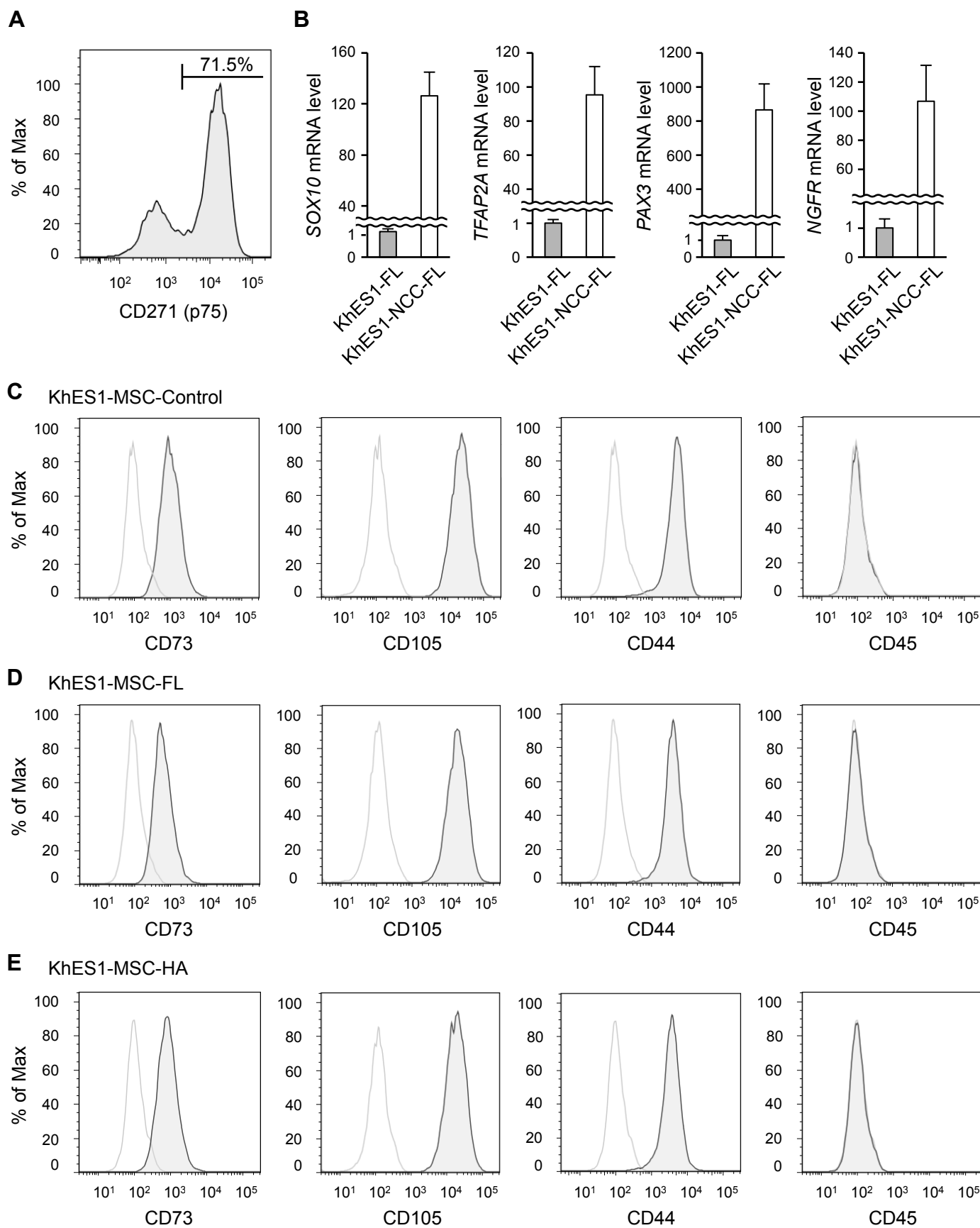

**S2 Fig. Characterization of KhES1-NCCs and KhES1-MSCs.**

Supplement: S2 Fig — A) Induction efficiency of NCCs from KhES1-FL cells. After the neural crest induction, cells were stained with an anti-p75 antibody and the p75high-positive population was analyzed by FACS. B) Expression of neural crest-specific markers in KhES1-FL and KhES1-NCC-FL cells. The mRNA expression of hNCC markers (SOX10, TFAP2A, PAX3, and NGFR) was analyzed by RT-qPCR in cells with SS18-SSX2 without the DOX treatment. Expression levels were normalized to those of human ACTB and calculated as fold changes relative to KhES1-FL cells. Error bars reflect SD in 3 experiments. C-E) Expression of surface markers in hMSC cells. After the induction of hMSCs, the expression of each CD antigen in KhES1-MSC-Control (C), KhES1-MSC-FL (D), and KhES1-MSC-HA (E) cells was analyzed by FACS. (PDF) [file pone.0142991.s002.pdf]

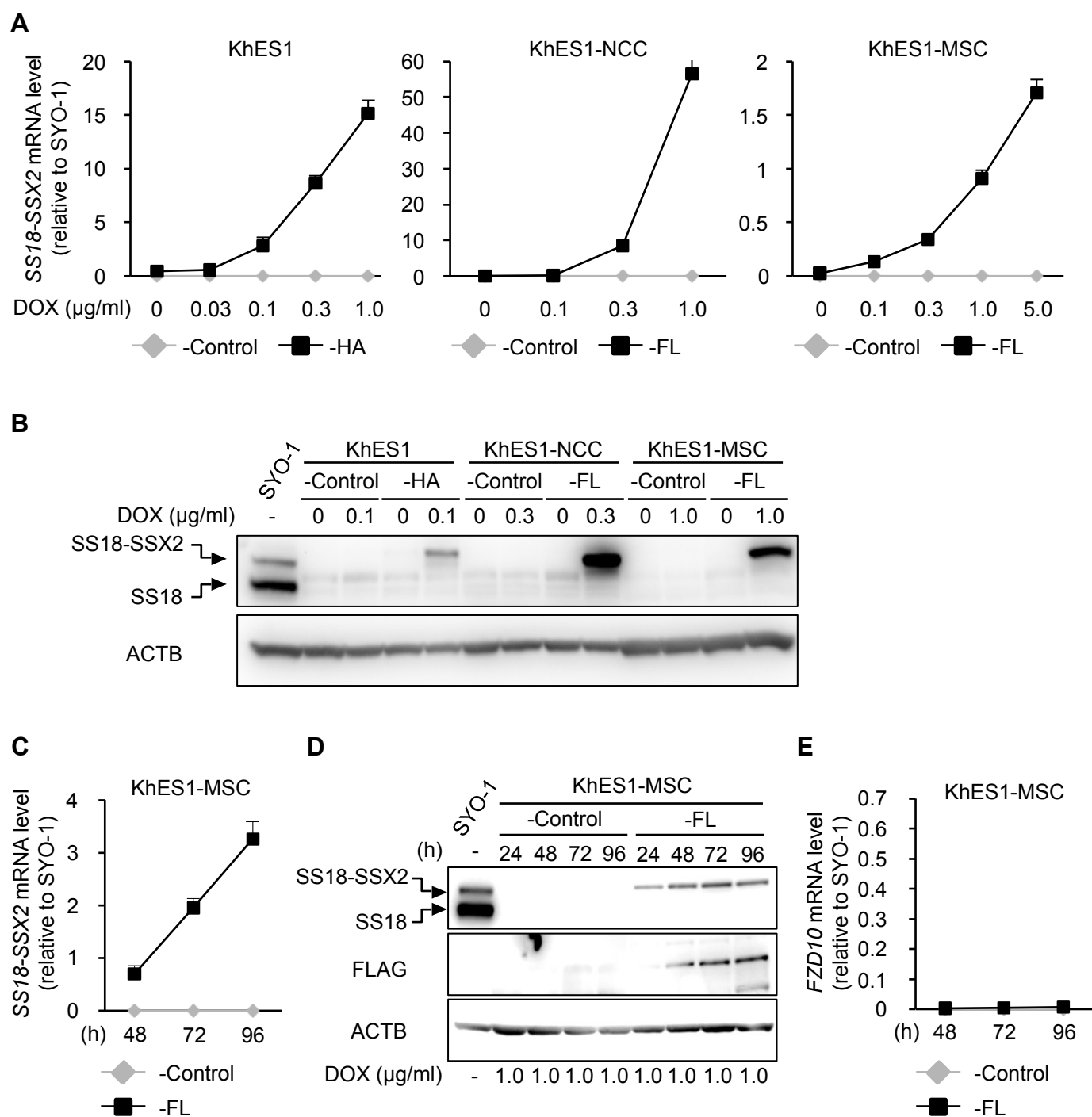

**S4 Fig. Induction of SS18-SSX2 in hESCs, hNCCs, and hNCC-derived MSCs.**

Supplement: S4 Fig — A) DOX dose-dependently induced SS18-SSX2 mRNA in KhES1-HA, KhES1-NCC-FL, and KhES1-MSC-FL cells. Cells with Stuffer (-Control) and SS18-SSX2 were treated with the indicated concentrations of DOX for 24 h, and the expression of SS18-SSX2 was analyzed by RT-qPCR. Expression levels were normalized to those of human ACTB and calculated as fold changes relative to SYO-1. Error bars reflect SD in 3 experiments. B) Comparison of SS18-SSX2 expression levels among KhES1-HA, KhES1-NCC-FL, and KhES1-MSC-FL cells. Cells with Stuffer (-Control) and SS18-SSX2 were treated with the indicated concentrations of DOX for 24 h, and the expression of SS18-SSX2 was analyzed by Western blotting. The SS18-SSX2 and SS18 proteins were detected using an anti-SS18 antibody. C and D) The time-dependent induction of SS18-SSX2 at mRNA (C) and protein (D) levels in KhES1-MSC-FL cells. Cells with Stuffer (-Control) and SS18-SSX2 were treated with 1.0 μg/ml of DOX for the indicated periods. C) RT-qPCR; Expression levels were normalized to those of human ACTB and calculated as fold changes relative to SYO-1. Error bars reflect SD in 3 experiments. D) Western blotting; The SS18-SSX2 and SS18 proteins were detected by an anti-SS18 antibody (top panel), and the FLAG-SS18-SSX2 protein was detected using an anti-FLAG antibody (middle panel). E) Induction of FZD10 expression by SS18-SSX2 in KhES1-MSC-FL cells. Cells with Stuffer (-Control) and SS18-SSX2 were treated with 1.0 μg/ml of DOX for the indicated periods. The expression of FZD10 was analyzed by RT-qPCR. Expression levels were normalized to those of human ACTB and calculated as fold changes relative to SYO-1. Error bars reflect SD in 3 experiments. (PDF) [file pone.0142991.s004.pdf]

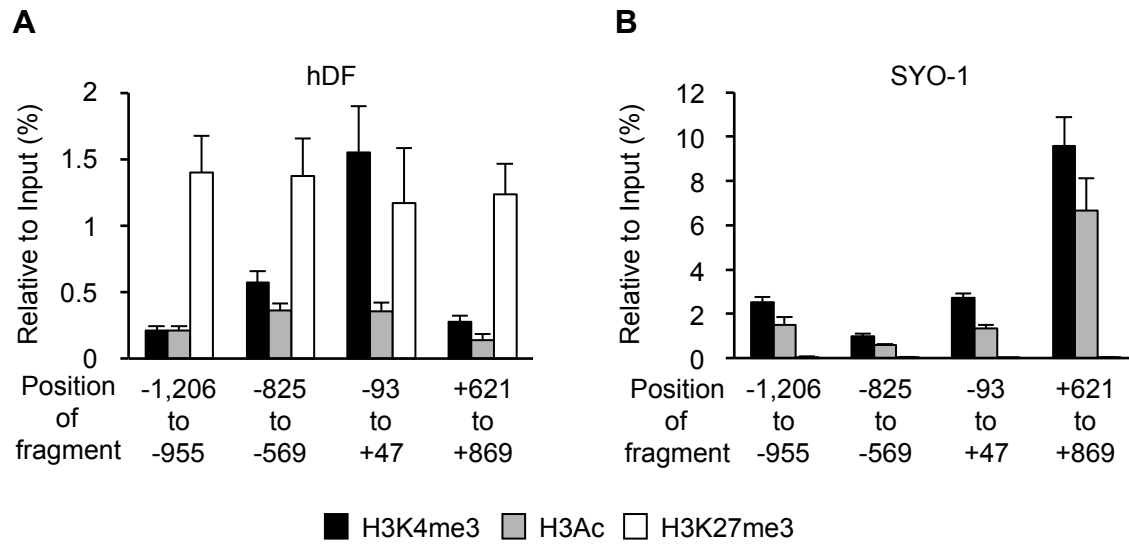

**S5 Fig. Histone modifications at the *FZD10* locus in fibroblasts and SS cells.**

Supplement: S5 Fig — A and B) Modifications of histones associated with 5’ regions in the FZD10 locus of hDF (A) and SYO-1 (B) cells. H3K4me3, H3Ac, and H3K27me3 levels were analyzed by ChIP-qPCR. The values indicate relative to the input. Error bars reflect SD in 3 experiments. (PDF) [file pone.0142991.s005.pdf]

**A**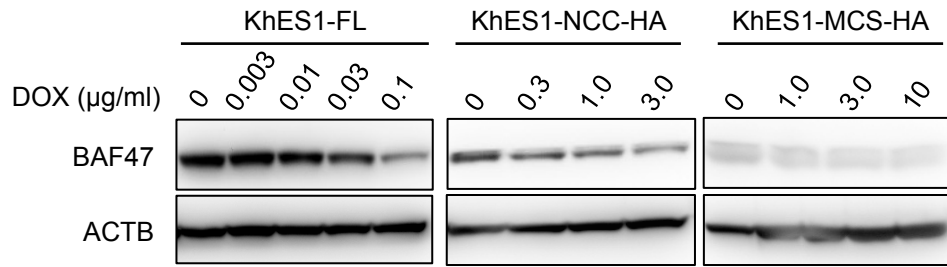**B**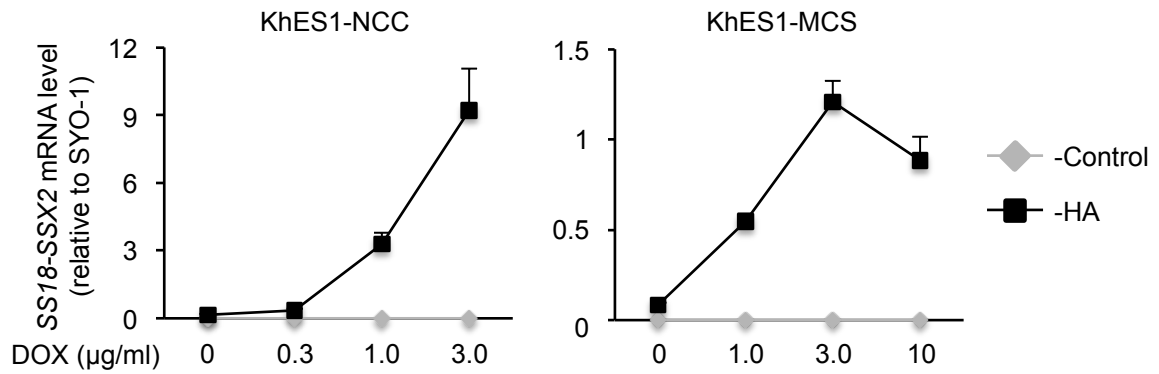**C**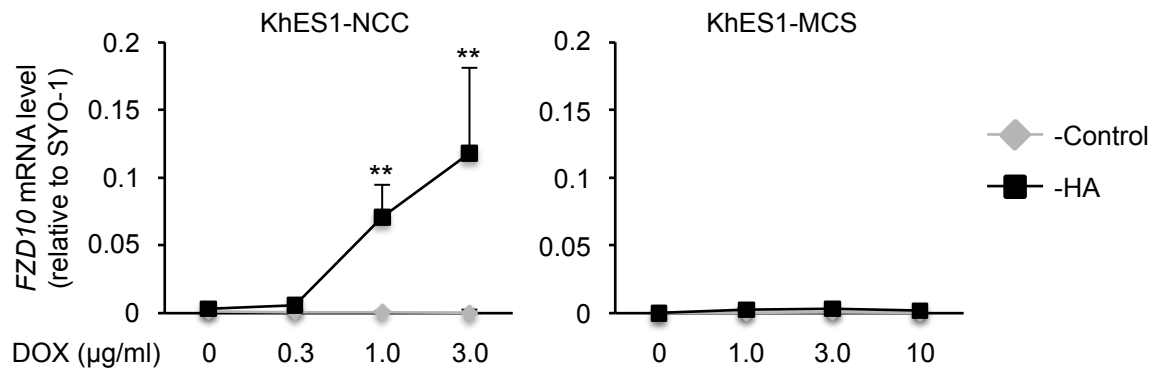

**S6 Fig. Relationship between BAF47 levels and the induction of *FZD10*.**

Supplement: S6 Fig — A) Effects of SS18-SSX2 on BAF47 expression levels. KhES1-FL, KhES1-NCC-HA, and KhES1-MSC-HA cells were treated with the indicated concentrations of DOX for 24 h, and the expression of BAF47 was analyzed by Western blotting. The BAF47 protein was detected using an anti-BAF47 antibody. B and C) Induction of SS18-SSX2 (B) and FZD10 (C) mRNA in KhES1-NCC-HA and KhES1-MSC-HA cells. Cells with Stuffer (-Control) and SS18-SSX2 were treated with the indicated concentrations of DOX for 24 h, and the expression of SS18-SSX2 (B) and FZD10 (C) was analyzed by RT-qPCR. Expression levels were normalized to those of human ACTB and calculated as fold changes relative to SYO-1. Error bars reflect SD in 3 experiments. Error bars reflect SD in 3 experiments. **, p<0.01 by the t-test. (PDF) [file pone.0142991.s006.pdf]
