# Supplementary material for: SS18-SSX, the Oncogenic Fusion Protein in Synovial Sarcoma, Is a Cellular Context-Dependent Epigenetic Modifier
Source: PLoS One. 2015 Nov 16;10(11):e0142991. doi: 10.1371/journal.pone.0142991 (PMC4646489; doi:10.1371/journal.pone.0142991)
Supplement: S3 Fig — A-C) KhES-MSC-Control, KhES1-MSC-FL, and KhES1-MSC-HA cells were induced toward osteogenic (A), chondrogenic (B), or adipogenic (C) lineages. Osteogenic induction (OI), chondrogenic induction (CI), and adipogenic induction (AI) were performed as described in the Materials and Methods section, and were evaluated by Alizarin Red staining on day 14, Alcian Blue staining on day 10, and Oil Red O staining on day 18, respectively. hMSCs were cultured during the induction periods in hMSC medium as a negative control (CT). Scale bar, 200 μm in OI and 50 μm in AI. (PDF) [file pone.0142991.s003.pdf]

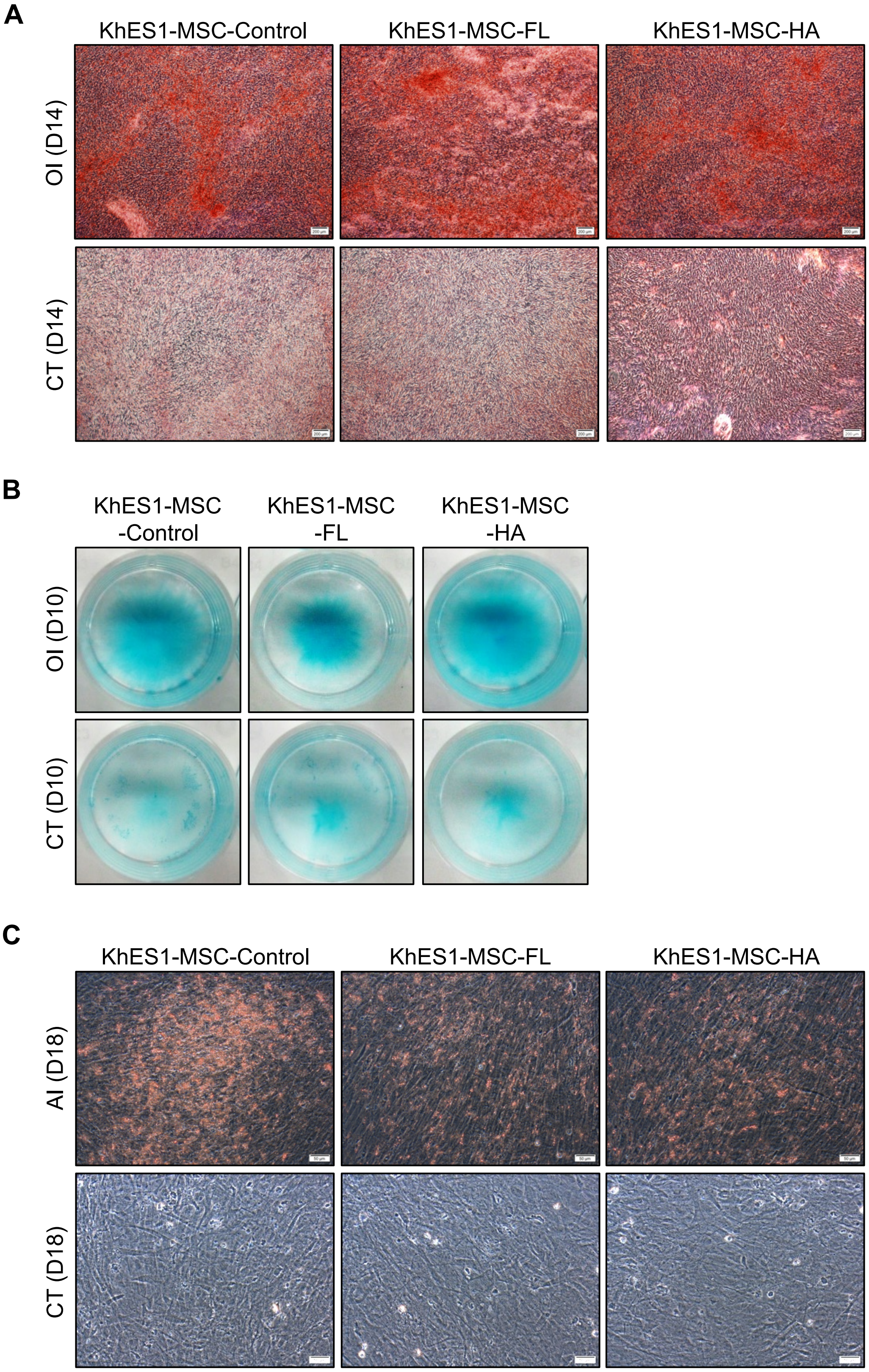

S3 Fig. Differentiation properties of KhES1-MSCs toward osteogenic, chondrogenic, and adipogenic lineages.
