# Supplementary material for: SS18-SSX, the Oncogenic Fusion Protein in Synovial Sarcoma, Is a Cellular Context-Dependent Epigenetic Modifier
Source: PLoS One. 2015 Nov 16;10(11):e0142991. doi: 10.1371/journal.pone.0142991 (PMC4646489; doi:10.1371/journal.pone.0142991)
Supplement: S1 Table — (PDF) [file pone.0142991.s007.pdf]

S1 Table. Primer and siRNA sequences used in this study.

Primer sets for constructions in the Luc assay

Name	Sequence (5'→3')
FZD10 (-1305) KPN-Sense	GTG GGTACC AAGCACCTGGCGCATCCTTG
FZD10 (-752) KPN-Sense	CCG GGTACC TTCCCGCCTTCTTAGAGGAG
FZD10 (-335) Xho I-Sense	CCG CTCGAG AGAGGCAAAGTTGGAGGCCA
FZD10 (-91) Xho I-Sense	CCG CTCGAG TTTCTCATGCAAAGCAGCG
FZD10 (-39) Xho I-Sense	CCG CTCGAG TGTCAGCGCTCGGCTTTTCT
FZD10 (+275) Hind III-Antisense	CCC AAGCTT AAGTTTGCAAACAATACCGGG

Primer sets for qPCR

Name	Conventional PCR and/or qPCR	Forward (5'→3')	Reverse (5'→3')
SS18-SSX1	Conventional PCR	CAACAGCAAGATGCATACCA	GGTGCAGTTGTTTCCCATCG
SS18-SSX2	Conventional PCR	CAACAGCAAGATGCATACCA	GGCACAGCTCTTTCCCATCA
SS18-SSX2	qPCR; SYBR GREEN	AGCAGAGGCCTTATGGATATGA	GGCACAGCTCTTTCCCATCA
FZD10	Both	TATCGGGCTCTTCTCTGTGC	GACTGGGCAGGGATCTCATA
SOX10	qPCR; SYBR GREEN	GAGCTGGACCGCACACCTTGGG	AACGCCCACCTCCTCGGACCTC
TFAP2A	qPCR; SYBR GREEN	AGGGCCTCGGTGAGATAGTT	AAGAGTTCACCGACCTGCTG
PAX3	qPCR; SYBR GREEN	CGGCATCCTGAGCGAGCGAG	ACTCGGGCCTCGGTGAGCTT
NGFR	qPCR; SYBR GREEN	CCGTTGGATTACACGGTCCA	GACAGGGATGAGGTTGTCGG
ACTB	Conventional PCR	AAGAGAGGCATCCTCACCCT	TACATGGCTGGGGTGTTGAA
ACTB	qPCR; SYBR GREEN	AGGTCTTTGCGGATGTCCACGT	CACCATTGGCAATGAGCGGTTC

Primer sets for ChIP-qPCR

Name	Forward (5'→3')	Reverse (5'→3')
FZD10 (-1206 to -955)	CTAAGTGACCCCGTCACAGACC	ATTTGGCGAGTGATGTGCCC
FZD10 (-825 to -569)	CAAACCAAGCCCGCACTTCC	GCCCTTGCTTGTAAACCTGCC
FZD10 (-93 to +47)	GCTTTCTCATGCAAAGCAGCGG	CAGCCGGCAGCTGTTTCGAGG
FZD10 (+621 to +869)	GCTACAACATGACTCGTATGCC	GCCACTTGAAGTTGAACTGCTCC

siRNAs against *SS18-SSX2*

	Sense (5'→3')	Antisence (5'→3')
si-*SS18-SSX2* #1	UGACCAGAUCAUGCCCAAGdTdT	CUUGGGCAUGAUCUGGUCAdTdT
