# Supplementary material for: SS18-SSX, the Oncogenic Fusion Protein in Synovial Sarcoma, Is a Cellular Context-Dependent Epigenetic Modifier
Source: PLoS One. 2015 Nov 16;10(11):e0142991. doi: 10.1371/journal.pone.0142991 (PMC4646489; doi:10.1371/journal.pone.0142991)
Supplement: S2 Table — (PDF) [file pone.0142991.s008.pdf]

S2 Table. Antibodies used in this study.

Antibodies for Western blotting

Name	Company	Catalog No.
SYT Antibody (H-80)	Santa Cruz	sc-28698
Anti-HA tag antibody - ChIP Grade	Abcam	ab9110
Monoclonal ANTI-FLAG M2-Peroxidase (HRP) antibody produced in the mouse	SIGMA	A8592
Ini1 (A5)	Santa Cruz	sc-166165
Monoclonal Anti-b-Actin-Peroxidase clone AC-15	SIGMA	A3854

Antibodies for ChIP

Name	Company	Catalog No.
Anti-HA tag antibody - ChIP Grade	Abcam	ab9110
Anti-trimethyl-Histone H3 (Lys4)	Millipore	07-473
ChIPAb+ Acetyl-Histone H3	Millipore	17-615
Anti-trimethyl-Histone H3 (Lys27)	Millipore	07-449

Antibodies for FACS

Name	Company	Catalog No.
p75(CD271)AF-647	BD	560326
CD73	BD	550257
CD105	eBioscience	17-1057-73
CD44	BD	555479
CD45	BD	340913
