# Supplementary material for: SS18-SSX, the Oncogenic Fusion Protein in Synovial Sarcoma, Is a Cellular Context-Dependent Epigenetic Modifier
Source: PLoS One. 2015 Nov 16;10(11):e0142991. doi: 10.1371/journal.pone.0142991 (PMC4646489; doi:10.1371/journal.pone.0142991)
Supplement: S3 Table — (PDF) [file pone.0142991.s009.pdf]

S3 Table. Cell lines and conditions for gene expression profiling.

Cell name described in Figure 3	Cell type	Inducible gene	Concentration of DOX (μg/ml)
hPSCs	KhES1	FLAG-SS18-SSX2	0
	414C2	FLAG-SS18-SSX2	0
hPSCs with SS18-SSX2	KhES1	FLAG-SS18-SSX2	0.03
	414C2	FLAG-SS18-SSX2	0.03
hPSC-NCCs	KhES1-NCC	Stuffer	0.3
	414C2-NCC	Stuffer	0.3
hPSC-NCCs with SS18-SSX2	KhES1-NCC	FLAG-SS18-SSX2	0.3
	414C2-NCC	FLAG-SS18-SSX2	0.3
hPSC-MSCs	KhES1-MSC	Stuffer	0.3
	414C2-MSC	Stuffer	0.3
hPSC-MSCs with SS18-SSX2	KhES1-MSC	FLAG-SS18-SSX2	0.3
	414C2-MSC	FLAG-SS18-SSX2	0.3
SS cell lines	SYO-1	None	-
	1273-99	None	-
	Fuji	None	-

N=1 for each sample.
